# Novel risk prediction models for deep vein thrombosis after thoracotomy and thoracoscopic lung cancer resections, involving coagulation and immune function

**DOI:** 10.1515/biol-2022-0617

**Published:** 2023-05-23

**Authors:** Jianhua Li, Futao Zhang, Xinyan Lan, Feifei Li, Chunrui Tan, Wangkai Cao

**Affiliations:** Department of Thoracic Surgery, Chengyang District People’s Hospital, Qingdao, Shandong, China; Weifang Second People’s Hospital, Weifang, Shandong, China; People’s Hospital of Jimo District, Qingdao, Shandong, China

**Keywords:** lung cancer resection, machine learning, prospective study, venous thrombosis, lung cancer

## Abstract

The main focus of this study was to compare the predictive value of coagulation, fibrinolysis, thromboelastography, stress response, and immune function in predicting the incidence of deep venous thrombosis (DVT) in lung cancer (LC) patients undergoing thoracoscopic LC resection vs thoracotomy LC resection. To do that, a prospective, single-center, case-control study involving 460 LC patients was conducted. The risk indicators affecting patients with DVT after LC resection in the testing cohort were determined using logistic regression and receiver operator characteristic (ROC) analyses. One validation cohort was used to assess the risk prediction models. DVT incidence was higher in the thoracoscopic group (18.7%) than in the thoracotomy group (11.2%) in the testing cohort (*χ* ^2^ = 4.116, *P* = 0.042). The final model to predict the incidence of DVT after thoracoscopic LC excision (1 day after surgery) was as follows: Logit(*P*) = 9.378 – 0.061(*R*-value) – 0.109(*K* value) + 0.374(α angle) + 0.403(MA) + 0.298(FIB) + 0.406(D-D) + 0.190(MDA) − 0.097(CD4^+^/CD8^+^). For thoracotomy LC resection, the final model (3 days after operation) was: Logit(*P*) = –2.463 − 0.026(*R*-value) − 0.143(*K* value) + 0.402(α angle) + 0.198(D-D) + 0.237(MDA) + 0.409(SOD). In the validation cohort, this risk prediction model continued to demonstrate good predictive performance. As a result, the predictive accuracy of postoperative DVT in patients who underwent thoracoscopic LC resection and thoracotomy LC resection was improved by risk prediction models.

## Graphical abstract

**Figure:**
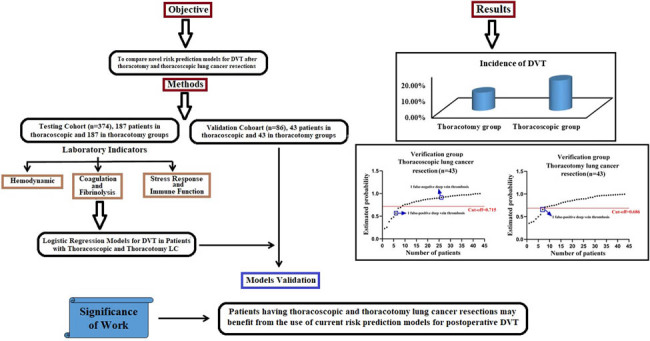

## 1 Introduction

Lung cancer (LC) is one of the most rapidly growing malignant tumors and poses the highest threat to human life and health in terms of morbidity and mortality [1]. The reported survival rate for patients diagnosed with LC at an early stage is over 90%; however, this rate decreases to less than 20% for those diagnosed at a later stage, which is directly correlated with tumor metastasis [2]. Patients with LC must continue to adhere to the treatment principles of early treatment to achieve the greatest clinical benefit. In the current standard LC treatment methodologies, surgical treatment can be divided into two categories based on the various surgical procedures, namely, thoracotomy LC resection and thoracoscopic LC resection.

Traditional thoracotomy LC resection has several benefits, including a distinct surgical field, a simple operation, an accurate resection range, and a generally low postoperative recurrence rate [3]. However, it has significant drawbacks, such as significant trauma, significant postoperative bleeding, and a strong postoperative stress response [3]. These factors will significantly hinder the recovery of LC patients following surgery. Patients who have undergone thoracotomy LC resection are susceptible to a variety of postoperative complications, such as incision infection and poor surgical incision healing, which further prolong the hospital stay [4]. Compared to thoracotomy LC resection, thoracoscopic surgery offers numerous benefits, including a smaller incision, less trauma, and less blood loss [5]. In addition, the gastrointestinal function of patients undergoing thoracoscopic operation recovers rapidly, facilitating the rapid reconstruction of enteral nutrition [5]. More significantly, thoracoscopic surgery can help to strengthen the body’s immune function and encourage patients to endure systemic chemotherapy or radiotherapy, resulting in greater clinical benefits [6].

With the widespread implementation of thoracoscopic operation and the accumulation of prognostic data, the closely related complications of thoracoscopic surgery have attracted people’s attention and are becoming an area of extensive investigation. Venous thromboembolism, which includes deep venous thrombosis (DVT) and pulmonary embolism, is a frequent postoperative complication associated with significant morbidity, mortality, and utilization of resources [7,8]. DVT is a disorder of venous return produced by abnormal clotting of blood in the deep veins of the lower extremities. According to studies, the incidence of DVT ranges from 10 to 40% [9,10]. The risk of DVT has been underestimated, although postoperative DVT is one of the more frequent complications in the perioperative phase of surgery. The low incidence of postoperative DVT observed in patients having thoracic surgery may be because perioperative or postoperative DVT can be asymptomatic [11]. The elevated disability and mortality rates associated with DVT necessitate serious consideration. This highlights the significance of both identifying at-risk patients and identifying those who have already acquired DVT. The results of a study comparing postoperative DVT incidence between thoracoscopic and thoracotomy LC resections are not yet known. To easily estimate DVT, we conducted a single-center prospective case-control study in this work to develop novel logistic regression models of thoracoscopic LC resection and thoracotomy LC resection based on the coagulation, fibrinolysis, thromboelastography, stress response, and immune function laboratory indicators. These models were then validated in one external validation cohort.

## 2 Materials and methods

### 2.1 General information on LC patients included in this study

From June 2017 to February 2019, a total of 602 LC patients diagnosed at the Chengyang District People’s Hospital were included in the study. Patients who met the inclusion and exclusion criteria of the study were evaluated for eligibility and then regularly contacted via telephone for follow-up. After excluding those who were lost to follow-up, 460 LC patients were enrolled in the study.


**Informed consent:** Informed consent has been obtained from all individuals included in this study.
**Ethical approval:** The research related to human use has been complied with all the relevant national regulations, institutional policies, and in accordance with the tenets of the Helsinki Declaration, and has been approved by Chengyang District People’s Hospital’s ethics committees (as revised in 2013).

### 2.2 Inclusion and exclusion criteria

The inclusion criteria for the patients were as follows: (1) The patient was over the age of 18. (2) Pathological examination confirmed the patient’s diagnosis of LC. The pathology of the gross specimen of the patient was also diagnosed as LC. (3) The patient has no obvious surgical contraindications. (4) Patients can undergo radical LC resection. (5) For a preliminary evaluation of the patient’s condition, imaging examinations such as enhanced chest CT were performed prior to the operation. (6) An informed consent document for the procedure was signed by the patient and his family.

Patients were excluded based on the following criteria: (1) Before the operation, the patient got preoperative neoadjuvant chemotherapy, molecular targeted therapy, and other related treatments. (2) Before the surgery, the patient was examined and discovered to have organ malignancies other than LC. (3) Before undergoing an operation, the patient was examined and it was discovered that LC had metastasized. (4) During the operation, it was discovered that the patient’s tumor had widespread metastasis or implant metastasis in the abdominal cavity. (5) Thoracoscopic LC resection was converted to thoracotomy. (6) The patient had previously undergone resection for LC, and he was diagnosed with residual LC at this time. (7) The preoperative anesthesia risk score was ≥3 grade. (8) Prior to an operation, the patient had a history of orally administered anticoagulants. (9) Contraindications for thoracoscopic operation include patients with abdominal wall hernia, severe cardiopulmonary disease before operation and cardiopulmonary function that cannot improve in a short time, coagulation dysfunction before operation, and a history of related diseases. (10) The patient or family members did not sign the preoperative informed consent.

### 2.3 Surgical procedure and follow-up

The thoracoscopic and traditional thoracotomy LC resections were performed as previously described [12]. All patients were followed up by telephone for 30 days after operation.

### 2.4 Observation and detection indicators

Patient demographics were recorded, including age, gender, height, weight, preoperative problems, pathological findings, and a history of any preexisting conditions such as hypertension, coronary heart disease, diabetes, and others. A patient’s anesthetic type, duration, operation time, intraoperative blood loss, and length of hospital stay following surgery were also recorded.

Thromboelastography was carried out as previously described [13]. The primary parameters of thromboelastography are the *K* value, reaction time (*R*), maximum amplitude (MA), and α angle [14]. The *K* value measures the rate of clot formation and is equal to the time required to produce thrombin. The time needed for the blood sample to identify the initial clot formation is represented by the *R*-value, which is equal to the thromboplastin generation time. When coagulation factors are absent or anticoagulants are present, the *R*-value is prolonged; in the hypercoagulable condition, it is shortened. The fibrinogen (FIB) thrombin and platelet concentrations, as well as their quality or amount, have a significant impact on the α angle, a parameter that measures the rate of clot formation. The rate of blood clot formation is indicated by both the *K* number and the α angle. Hypercoagulability is indicated by abnormalities in any of the coagulation parameters (lower *R* value or *K* value, larger α angle, greater MA value) [15]. Principal indices for evaluating coagulation and fibrinolysis: Before surgery and 1 day, 3 days, and 5 days after surgery, a number of coagulation parameters, including prothrombin time (PT), activated partial thromboplastin time (APTT), FIB, and D-dimer (D-D), were measured in both groups. A prothrombin international normalized ratio (INR) calculation was made to determine the INR shift [16].

Identification of stress response and cellular immune function markers: 4 mL of fasting venous blood from the patient was drawn and collected in tubes A and B on 1, 3, and 5 days after the operation. Tube A was centrifuged at 720 × *g* for 10 min in preparation for the test, and the supernatant was kept at −20°C. ELISA (kit purchased from Shanghai Enzyme-linked Biotechnology Co., Ltd) was used to determine the concentrations of malondialdehyde (MDA), superoxide dismutase (SOD), interleukin 6 (IL-6), and C-reactive protein (CRP) in the blood [17]. The FACSCalibur flow cytometer (BD Company, USA) was used to detect CD4^+^ and CD8^+^ in tube B and to calculate the percentage of CD4^+^/CD8^+^ [18].

### 2.5 Testing cohort

Using logistic regression on the testing population, prediction models for DVT have been developed. The population tested comprised 374 individuals with LC. Using a random number method, patients were assigned to either thoracoscopic or open thoracotomy LC resection.

### 2.6 Developing the logistic regression model’s formula

The model for predicting DVT has the following formula: Logit (*P* = DVT) = *X*_0_ + *X*_1_ *Y*_1_ + *X*_2_ *Y*_2_ + … + *X*_*n*_ *Y*_*n*_ = ln[*p*/(1 − *p*)], In this equation, “*p*” represents the estimated probability of DVT, “*X*” is the coefficient, and “*Y*” represents each indication.

### 2.7 Validation cohort

In a validation cohort, models of logistic regression prediction accuracy were evaluated and found to be accurate. The validation cohort for the present study consisted of 86 LC patients. Using a random number table, the 86 patients were equally divided into two groups: thoracoscopic (43 patients) and open (43 patients).

### 2.8 Experimental specimen collection, testing, and quality control principles

The venous blood collection method was utilized to obtain blood samples from both patient groups. Blood was obtained from both patient groups from the elbow vein. Blood samples were drawn at the same time from both groups of LC patients. Before operation and 1 day, 3 days, and 5 days after the operation, cubital venous blood was collected while the patient was fasting. The blood was drawn into the usual tube and the heparin-containing negative pressure tube for testing. The plasma was isolated for detection from the blood in the anticoagulation tube after centrifuging it for 20 min at 4°C in a refrigerator and 15 min at 3,000 rpm/centrifugal speed. The diagnostic equipment used on the two blood sample collections was identical. During the transportation and testing process, the same set of samples was tested at the same time, the experimental procedure was identical, and the testing was conducted by the same physician.

### 2.9 Statistical analysis

All experiments were performed in triplicate unless specified. The data were summarized using measures of central tendency and dispersion, such as the mean value ± standard deviation (SD) or the median and interquartile range (IQR) or the percentage. The Student’s *t*-test was used to compare normally distributed numeric variables, while the Mann–Whitney *U*-test was used to compare non-normally distributed variables. One-way analysis of variance (ANOVA) was used to compare groups where the variance was comparable, and the Kruskal–Wallis test was used to assess variables with non-normal distributions. The SNK analysis was utilized to compare the relevant categories. Receiver operator characteristic (ROC) curves were used to evaluate the efficacy of each indicator in detecting DVT by calculating the area under the ROC curve (AUC) and the 95% confidence interval (CI). All significant variables from the univariate study were included in the multivariate analysis of patients with post-operative DVT using a binary logistic regression model. All statistical testing was performed with SPSS version 22.0 (IBM Corporation). If the significance level is less than 0.05, the difference is statistically significant [19].

## 3 Results

### 3.1 Technical route

**Figure 1:**
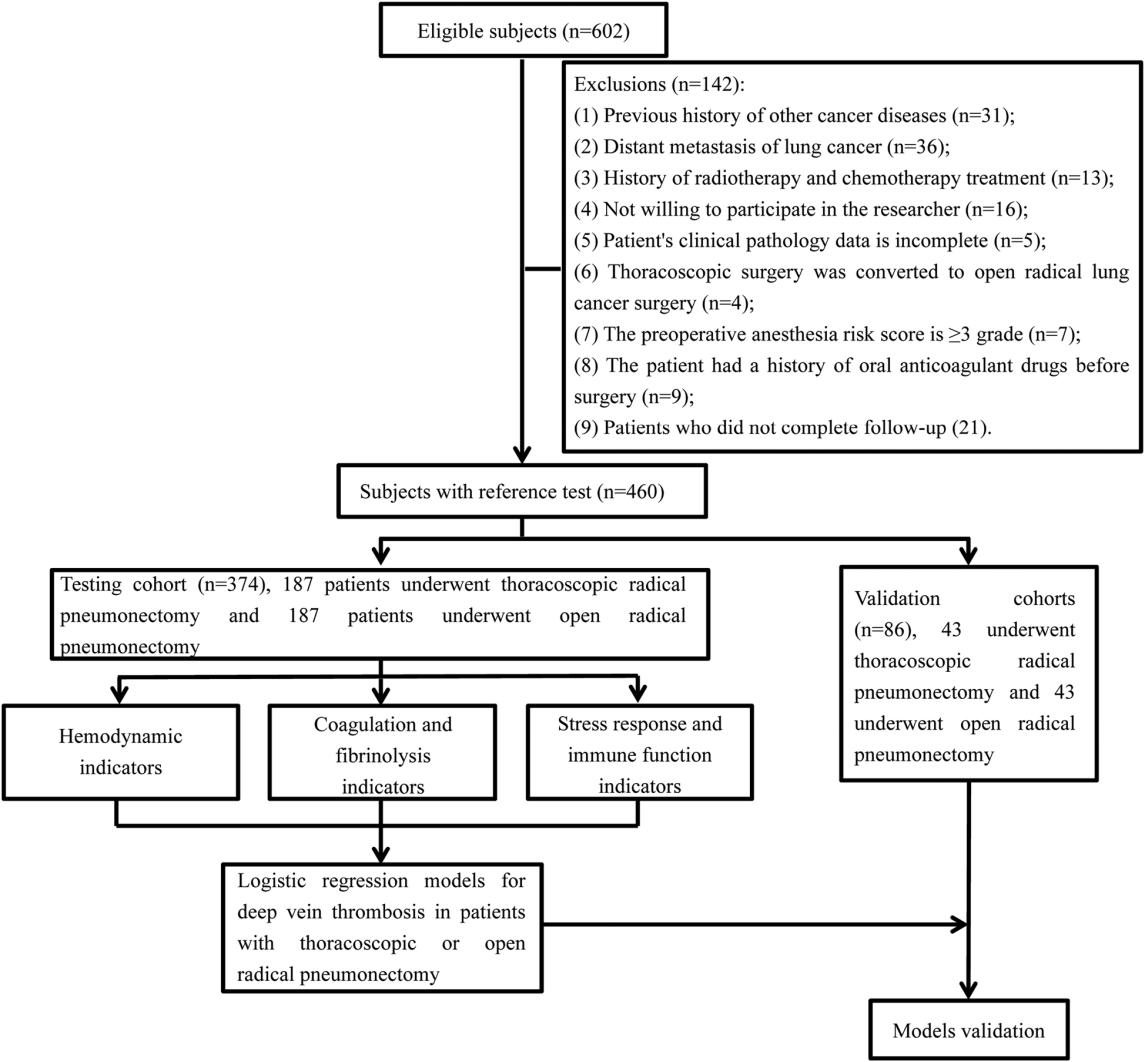
The technical route of this study.

Figure 1 depicts the technical methodology of this investigation. The inclusion criteria were not met by 142 out of 602 LC patients, so they were excluded. The included 460 patients were randomly assigned to the testing cohort and the verification cohort. Basic information of patients in thoracoscopic LC resection group (*n* = 187) and laparotomy group (*n* = 187) in the testing cohort and thoracoscopic group (*n* = 43) and laparotomy group (*n* = 43) in the verification cohort is shown in Table 1.

**Table 1:** Comparison of patients’ general clinical indicators and surgery-related indicators (results are represented as the mean values ± SD or median [interquartile range; IQR] or percentage)

Indicators	Testing cohort (*n* = 374)				Verification cohort (*n* = 86)			
	Thoracoscopic LC resection (*n* = 187)	Thoracotomy LC resection (*n* = 187)	*t*/*Z*/*χ* ^2^	*P*	Thoracoscopic LC resection (*n* = 43)	Thoracotomy LC resection (*n* = 43)	*t*/*Z*/*χ* ^2^	*P*
Gender (male/female)	126/61	137/50	1.550	0.213	32/11	29/14	0.508	0.476
Age	54 (26–78)	56 (29–77)	1.143	0.438	50 (25–73)	55 (31–75)	1.643	0.229
BMI (kg/m^2^)	24.11 ± 3.40	23.76 ± 3.04	1.206	0.228	23.91 ± 3.20	24.01 ± 3.34	−0.143	0.887
Hypertension (*n*, %)	49 (26.2)	43 (23.0)	0.519	0.471	9 (20.9)	7 (16.3)	0.307	0.579
Diabetes (*n*, %)	28 (15.0)	22 (11.8)	0.831	0.362	6 (14.0)	7 (16.3)	0.091	0.763
Hyperlipidemia (*n*, %)	31 (16.6)	26 (13.9)	0.517	0.472	8 (18.6)	10 (23.3)	0.281	0.596
Anticoagulation immediately after operation (*n*, %)	92 (49.2)	87 (46.5)	0.268	0.605	26 (60.5)	24 (55.8)	0.191	0.662
Tumor size (cm)	4.1 (1.2–7.3)	4.4 (1.1–7.6)	0.898	0.713	3.9 (0.9–6.9)	4.5 (1.0–7.7)	1.694	0.177
TNM staging (Ⅰ/Ⅱ/Ⅲ)	63/34/90	57/42/88	1.165	0.559	14/7/22	13/11/19	1.145	0.564
Operation time (min)	238.61 ± 36.53	195.90 ± 25.14	13.182	<0.001	226.42 ± 42.24	186.79 ± 30.71	4.976	<0.001
Intraoperative blood loss (mL)	188.83 ± 26.64	296.22 ± 80.8	−17.265	<0.001	175.73 ± 29.01	291.58 ± 77.42	−9.195	<0.001
Postoperative hospital stays (days)	9.71 ± 1.81	14.64 ± 2.30	−22.943	<0.001	9.32 ± 1.63	13.70 ± 2.46	−10.003	<0.001

Abbreviation: BMI: body mass index.

### 3.2 Comparison and analysis of laboratory indicators for coagulation, fibrinolysis, thromboelastography, stress response, and immune function between the thoracoscopic and thoracotomy LC resection groups in the testing cohort

The results of a comparison between the two groups using laboratory markers of coagulation, fibrinolysis, thromboelastography, stress response, and immunological function at different time intervals are presented in Table 2. There was no statistically significant difference between the two groups, despite the fact that both had comparable INR values before and after operation (all *P* > 0.05). The levels of the other markers, however, showed statistically significant changes at 1, 3, and 5 days after operation when compared to the same group prior to the procedure (all *P* < 0.05). Additionally, a pairwise comparison between groups was conducted. The thromoelastographic parameters of patients undergoing thoracoscopic LC resection compared to those undergoing thoracotomy LC resection differed significantly. Interestingly, we discovered that the most significant changes in the patients’ indicators occurred on day 1 after surgery in the thoracoscopic LC resection group, and on day 3 after surgery in the thoracotomy LC resection group (all *P* < 0.05). DVT incidence in LC was found to be associated with a shorter *K*-time, as well as a higher angle and MA [13]. In addition, MA, a well-known diagnostic marker for determining the risk of DVT, was most closely correlated with tumor stage, which reflects the contributions of platelet function and FIB deposition. As compared to the thoracotomy LC resection group at 3 days after surgery, *K*-time was considerably shorter and angle and MA were significantly higher in the study’s thoracoscopic LC resection group at 1 day after surgery. The result was highly correlated with previous investigations that identified LC using comparable values [20].

**Table 2:** Comparison of changes in thromboelastography, coagulation, fibrinolysis, stress response, and immune function indicators at different time points before and after operation in the testing cohort (*n* = 374) (results are represented as the mean values ± SD)

Indicators	Thoracoscopic LC resection (*n* = 187)				Thoracotomy LC resection (*n* = 187)			
	Preoperative	1 day after operation	3 days after operation	5 days after operation	Preoperative	1 day after operation	3 days after operation	5 days after operation
**Thromboelastography indicators**								
*R* value (min)	5.68 ± 1.17	4.87 ± 0.97^a^	5.34 ± 1.06^a,b^	5.39 ± 1.11^a,b^	5.69 ± 1.28	5.31 ± 1.15^a^	5.07 ± 1.08^a,b^	5.27 ± 1.13^a,c^
*K* value (min)	1.64 ± 0.50	1.19 ± 0.37^a^	1.47 ± 0.45^a,b^	1.59 ± 0.49^b,c^	1.61 ± 0.53	1.37 ± 0.42^a^	1.22 ± 0.31^a,b^	1.41 ± 0.44^a,c^
α angle (degree)	63.14 ± 5.06	77.18 ± 6.67^a^	73.27 ± 5.49^a,b^	71.50 ± 5.65^a,b,c^	63.17 ± 4.98	67.11 ± 5.11^a^	74.34 ± 5.89^a,b^	69.54 ± 6.03^a,b,c^
MA (mm)	66.19 ± 7.11	75.63 ± 7.33^a^	70.95 ± 7.13^a,b^	70.88 ± 7.46^a,b^	66.15 ± 7.24	70.05 ± 7.75^a^	76.83 ± 8.27^a,b^	69.87 ± 8.04^a,c^
**Coagulation indicators**								
PT (s)	12.71 ± 1.22	10.84 ± 0.91^a^	12.04 ± 1.11^a,b^	12.73 ± 1.10^b,c^	12.68 ± 1.20	12.64 ± 1.11	10.68 ± 0.94^a,b^	11.93 ± 1.05^a,b,c^
APTT (s)	30.11 ± 4.11	29.27 ± 4.08^a^	29.99 ± 4.18^a,b^	30.07 ± 3.89^b^	30.15 ± 4.15	29.94 ± 4.02^a^	29.48 ± 4.16^a,b^	29.90 ± 4.11^a,c^
INR	1.01 ± 0.09	1.03 ± 0.06	1.02 ± 0.10	1.00 ± 0.09	1.03 ± 0.11	1.01 ± 0.12	1.02 ± 0.09	1.01 ± 0.13
FIB (g/L)	2.74 ± 0.42	4.48 ± 0.57^a^	3.84 ± 0.32^a,b^	3.79 ± 0.49^a,b^	2.78 ± 0.45	3.47 ± 0.46^a^	4.36 ± 0.53^a,b^	4.18 ± 0.39^a,b,c^
D-D (mg/L)	0.15 ± 0.05	0.75 ± 0.23^a^	0.42 ± 0.11^a,b^	0.35 ± 0.09^a,b,c^	0.14 ± 0.04	0.29 ± 0.07^a^	0.81 ± 0.16^a,b^	0.46 ± 0.13^a,b,c^
**Stress response indicators**								
MDA (mmol/mL)	4.25 ± 1.03	7.42 ± 1.26^a^	5.87 ± 1.14^a,b^	5.09 ± 1.08^a,b,c^	4.21 ± 0.97	5.94 ± 1.02^a^	8.87 ± 1.24^a,b^	6.77 ± 1.11^a,c^
SOD (U/mL)	87.56 ± 11.07	67.25 ± 8.86^a^	75.15 ± 10.80^a,b^	86.98 ± 10.86^b,c^	87.47 ± 10.85	74.10 ± 9.37^a^	64.20 ± 7.97^a,b^	71.34 ± 9.11^a,c^
IL-6 (pg/mL)	8.52 ± 1.24	37.03 ± 9.05^a^	22.09 ± 5.38^a,b^	15.98 ± 5.89^a,b,c^	8.54 ± 1.22	19.87 ± 5.44^a^	35.12 ± 8.65^a,b^	23.15 ± 6.87^a,c^
CRP (ng/mL)	11.94 ± 2.24	39.21 ± 7.69^a^	18.40 ± 5.26^a,b^	18.33 ± 5.08^a,b^	11.90 ± 2.16	17.50 ± 4.82^a^	34.72 ± 6.97^a,b^	27.09 ± 5.42^a,c^
**Immune function indicators**								
CD4^+^ (%)	44.57 ± 4.06	22.89 ± 4.82^a^	27.05 ± 4.96^a,b^	29.76 ± 5.03^a,b,c^	44.16 ± 3.89	28.19 ± 4.48^a^	23.17 ± 4.56^a,b^	28.20 ± 4.87^a,c^
CD8^+^ (%)	29.04 ± 2.78	17.36 ± 2.36^a^	18.04 ± 3.16^a,b^	19.54 ± 2.61^a,b,c^	29.11 ± 2.54	18.11 ± 3.03^a^	17.64 ± 2.51^a,b^	18.77 ± 2.54^a,b,c^
CD4^+^/CD8^+^	1.53 ± 0.18	1.32 ± 0.14^a^	1.50 ± 0.13^b^	1.51 ± 0.16^b^	1.52 ± 0.15	1.56 ± 0.11	1.31 ± 0.15^a,b^	1.50 ± 0.14^c^

**Abbreviation:** *R* value: reaction time value; MA: maximum amplitude; PT: prothrombin time; APTT: activated partial thromboplastin time; FIB: fibrinogen; D-D: D-dimerization; IL-6: interleukin 6; MDA: malondialdehyde; SOD: superoxide dismutase; CRP: C-reactive protein.

### 3.3 Comparison of the DVT risk between the thoracoscopic and thoracotomy LC resection groups in the testing cohort

DVT occurred in 35% of patients who underwent thoracoscopic LC resection and in 21% of patients who underwent thoracotomy LC resection. In the testing cohort of patients having LC resection, the incidence of DVT was higher in the thoracoscopic group (18.7%) compared to the thoracotomy group (11.2%; *χ* ^2^ = 4.116, *P* = 0.042).

### 3.4 Comparison of coagulation, fibrinolysis, thromboelastography, stress response, and immune function laboratory markers between DVT and non-DVT groups

Table S1 shows the comparison between the DVT group and the non-DVT group based on the indicators at various time points, such as 1 day and 3 days following the procedure. It was discovered that the markers changed most significantly in the thoracoscopic LC resection group 1 day after surgery and in the thoracotomy LC resection group 3 days after surgery (all *P* < 0.05). Additionally, the DVT group had a reduced operating time, less blood loss during surgery, and a shorter hospital stay following surgery than the non-DVT group (all *P* < 0.05).

### 3.5 Logistic regression and ROC analyses of coagulation, fibrinolysis, thromboelastography, stress response, and immune function laboratory indicators in testing cohort

Univariate and multivariate logistic regression analyses were performed to better understand the factors that contributed to the substantially increased risk of DVT in the thoracoscopic group compared to the thoracotomy group. The results of logistic regression analysis are displayed in Tables 3 and 4. 1 day after surgery, the levels of *R* value, *K* value, angle, MA, FIB, D-D, MDA, and CD4^+^/CD8^+^ were independent predictors of DVT after thoracoscopic LC resection. Three days after a thoracotomy LC resection, risk variables for DVT included *R* value, *K* value, angle, D-D, MDA, and SOD values.

**Table 3:** Univariate and multivariate logistic regression analyses of DVT in patients undergoing thoracoscopic LC resection

	Univariate model ^a^					Multivariate model ^a,b^				
Indicators	B	SE	*χ* ^2^	OR (95% CI)	*P*	β	Standardized β	*χ* ^2^	OR (95% CI)	*P*
Operation time (min)	0.136	0.109	1.557	0.842 (0.925–1.419)	0.146					
Intraoperative blood loss (mL)	0.023	0.124	0.034	1.023 (0.802–1.305)	0.853					
*R* value (min), 1 day after operation	−0.253	0.089	8.081	0.776 (0.652–0.924)	0.004	−0.061	0.020	9.303	0.941 (0.905–0.978)	0.002
*K* value (min), 1 day after operation	−0.198	0.072	7.563	0.820 (0.712–0.945)	0.006	−0.109	0.041	7.068	0.897 (0.827–0.972)	0.008
α angle (degree), 1 day after operation	0.178	0.054	10.866	1.195 (1.075–1.328)	0.001	0.374	0.106	12.449	1.454 (1.181–1.789)	<0.001
MA (mm), 1 day after operation	0.194	0.038	26.064	1.214 (1.127–1.308)	<0.001	0.403	0.098	16.911	1.496 (1.235–1.813)	<0.001
PT (s), 1 day after operation	0.202	0.118	2.930	1.224 (0.971–1.542)	0.087					
FIB (g/L), 1 day after operation	0.487	0.219	4.945	1.627 (1.059–2.500)	0.026	0.298	0.112	7.079	1.347 (1.082–1.678)	0.008
D-D (mg/L), 1 day after operation	0.385	0.177	4.731	1.470 (1.039–2.079)	0.030	0.406	0.135	4.873	1.347 (1.034–1.755)	0.027
MDA (mmol/mL), 1 day after operation	0.359	0.144	6.181	1.430 (1.079–1.897)	0.013	0.190	0.086	12.007	1.347 (1.138–1.594)	0.001
IL-6 (pg/mL), 1 day after operation	0.292	0.163	3.209	1.339 (0.973–1.843)	0.073					
CRP (ng/mL), 1 day after operation	0.158	0.077	4.210	1.171 (1.007–1.362)	0.040	0.503	0.167	3.184	1.347 (0.971–1.869)	0.074
CD4^+^/CD8^+^, 1 day after operation	−0.099	0.045	4.840	0.906 (0.829–0.989)	0.028	−0.097	0.038	6.516	0.908 (0.842–0.978)	0.011

**Abbreviation:** *R* value: reaction time value; MA: maximum amplitude; PT: prothrombin time; FIB: fibrinogen; D-D: D-dimerization; IL-6: interleukin 6; MDA: malondialdehyde; CRP: C-reactive protein; OR: odds ratio; CI: confidence interval.

^a^ The odds ratio corresponds to a unit increase in the variable; ^b^ The odds ratio was adjusted for all significant variables of the univariate logistic regression analysis.

**Table 4:** Univariate and multivariate logistic regression analyses of DVT in patients undergoing thoracotomy LC resection

	Univariate model^a^					Multivariate model^a,b^				
Indicators	B	SE	*χ* ^2^	OR (95% CI)	*P*	*β*	Standardized *β*	*χ* ^2^	OR (95% CI)	*P*
*R* value (min), 3 days after operation	−0.029	0.009	10.383	0.971 (0.954–0.989)	0.001	−0.026	0.012	4.694	0.974 (0.952–0.998)	0.030
*K* value (min), 3 days after operation	−0.117	0.041	8.143	0.890 (0.821–0.964)	0.004	−0.143	0.037	14.937	0.869 (0.806–0.932)	<0.001
α angle (degree), 3 days after operation	0.274	0.104	6.941	1.315 (1.073–1.613)	0.008	0.402	0.143	7.903	1.495 (1.129–1.978)	0.005
MA (mm), 3 days after operation	0.301	0.092	10.704	1.351 (1.128–1.618)	0.001	0.289	0.166	3.031	1.335 (0.964–1.848)	0.082
PT (s), 3 days after operation	−0.069	0.111	0.386	0.933 (0.751–1.160)	0.534					
FIB (g/L), 3 days after operation	0.271	0.132	4.215	1.311 (1.012–1.698)	0.040	0.472	0.253	3.481	1.603 (0.976–2.632)	0.062
D-D (mg/L), 3 days after operation	0.149	0.037	16.217	1.161 (1.079–1.248)	<0.001	0.198	0.087	5.180	1.219 (1.028–1.446)	0.023
MDA (mmol/mL), 3 days after operation	0.239	0.087	7.547	1.270 (1.071–1.506)	0.006	0.237	0.074	10.257	1.267 (1.096–1.465)	0.001
SOD (U/mL), 3 days after operation	0.354	0.083	18.191	1.425 (1.211–1.676)	<0.001	0.409	0.136	9.044	1.505 (1.153–1.965)	0.003
IL-6 (pg/mL), 3 days after operation	0.462	0.170	7.386	1.587 (1.137–2.215)	0.007	0.114	0.273	0.175	1.121 (0.656–1.914)	0.676

**Abbreviation:** *R* value: reaction time value; MA: maximum amplitude; PT: prothrombin time; FIB: fibrinogen; D-D: D-dimerization; IL-6: interleukin 6; MDA: malondialdehyde; SOD: superoxide dismutase; OR: odds ratio; CI: confidence interval.

^a^ The odds ratio corresponds to a unit increase in the variable; ^b^ The odds ratio was adjusted for all significant variables of the univariate logistic regression analysis.

**Figure 2:**
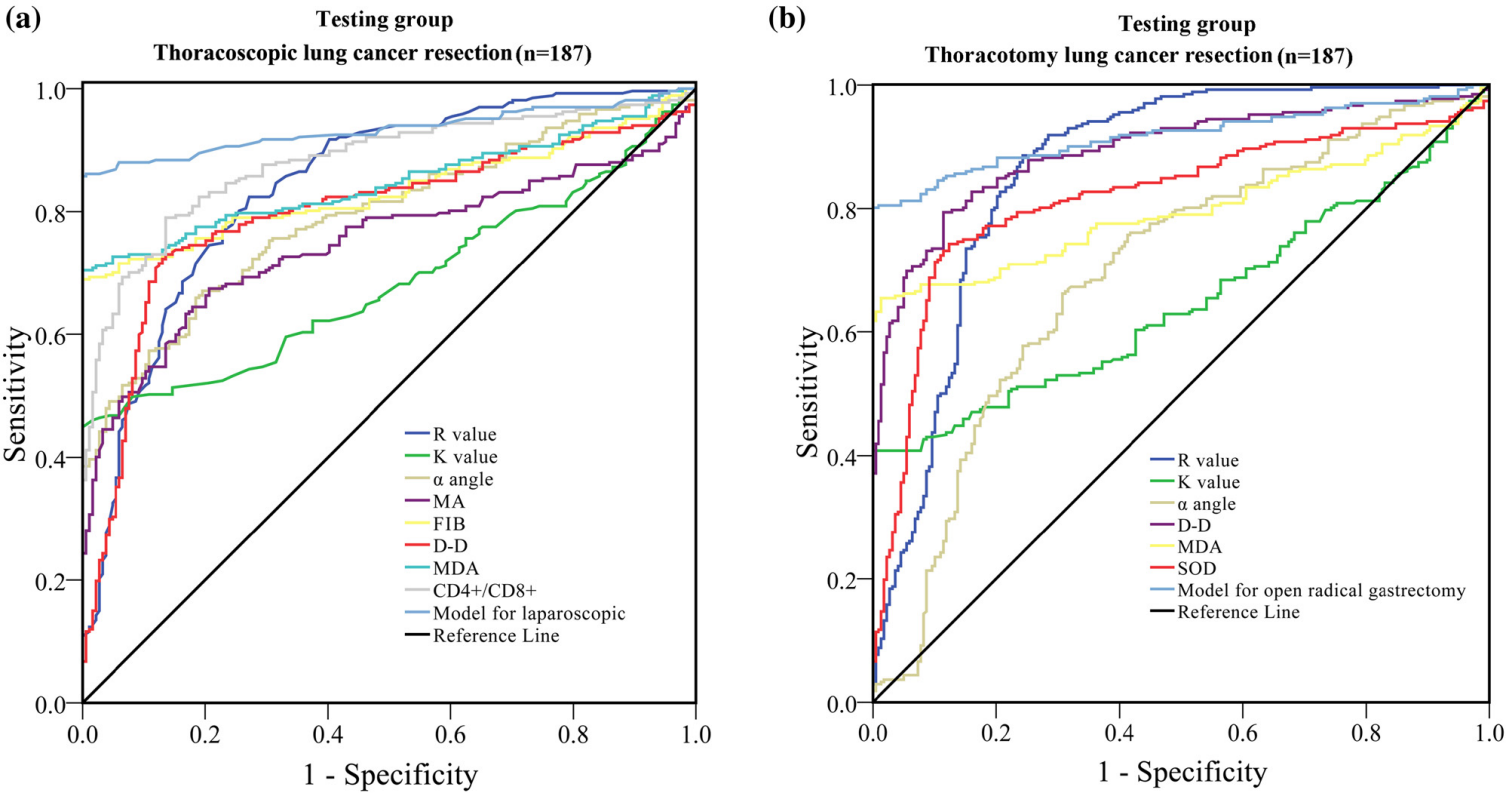
ROC analysis of coagulation, fibrinolysis, thromboelastography, stress response, and immune function laboratory indicators to predict the occurrence of DVT after LC resection in the testing cohort. (a) Thoracoscopic LC resection. (b) Thoracotomy LC resection. ROC: receiver operator characteristic; DVT: deep venous thrombosis.

The risk variables for DVT following LC surgery were established through multivariate logistic regression studies. Subsequently, these factors were subjected to further analysis using the ROC approach. The AUCs for the *R* value, *K* value, angle, MA, FIB, D-D, MDA, and CD4^+^/CD8^+^ in thoracoscopic LC resection were 0.843, 0.684, 0.792, 0.747, 0.835, 0.797, 0.848, and 0.881, respectively (Figure 2 and Table 5). The AUCs for the *R* value, *K* value, angle, D-D, MDA, and SOD in thoracotomy LC resection were 0.864, 0.649, 0.893, 0.796, 0.817, and 0.697 respectively.

**Table 5:** Calculated performance values for different indicators in the testing cohort

Variable	AUC	95% CI	*P*	Sensitivity (%)	Specificity (%)
**Thoracoscopic** LC **resection**					
*R* value (min), 1 day after operation	0.843	0.805–0.880	<0.001	90.1	62.3
*K* value (min), 1 day after operation	0.684	0.635–0.732	<0.001	52.4	93.4
α angle (degree), 1 day after operation	0.792	0.751–0.832	<0.001	77.8	66.2
MA (mm), 1 day after operation	0.747	0.702–0.792	<0.001	68.6	78.5
FIB (g/L), 1 days after operation	0.835	0.798–0.873	<0.001	68.8	98.6
D-D (mg/L), 1 day after operation	0.797	0.754–0.840	<0.001	72.9	86.7
MDA (mmol/mL), 1 day after operation	0.848	0.812–0.885	<0.001	54.1	91.3
CD4^+^/CD8^+^, 1 day after operation	0.881	0.849–0.912	<0.001	79.2	86.3
Model for thoracoscopic	0.934	0.909–0.958	<0.001	87.8	100.0
**Thoracotomy** LC **resection**					
*R* value (min), 3 days after operation	0.864	0.829–0.899	<0.001	90.2	71.3
*K* value (min), 3 days after operation	0.649	0.600–0.697	<0.001	42.3	100.0
α angle (degree), 3 days after operation	0.697	0.649–0.744	<0.001	68.9	67.8
D-D (mg/L), 3 days after operation	0.893	0.864–0.922	<0.001	82.2	86.3
MDA (mmol/mL), 3 days after operation	0.796	0.755–0.836	<0.001	67.9	97.5
SOD (U/mL), 3 days after operation	0.817	0.778–0.857	<0.001	73.6	89.2
Model for thoracotomy LC resection	0.918	0.892–0.944	<0.001	80.7	100.0

**Abbreviation:** *R* value: reaction time value; MA: maximum amplitude; PT: prothrombin time; FIB: fibrinogen; D-D: D-dimerization; MDA: malondialdehyde; SOD: superoxide dismutase; AUC: area under curve; OR: odds ratio; CI: confidence interval.

### 3.6 Novel risk prediction models for DVT based on the testing cohort

The aforementioned laboratory parameters were included in logistic regression models. The final model for predicting the incidence of DVT following thoracoscopic LC resection (1 day after surgery) was Logit(*P*) = 9.378 − 0.061(*R* value) − 0.109(*K* value) + 0.374(α angle) + 0.403(MA) + 0.298(FIB) + 0.406(D-D) + 0.190(MDA) – 0.097(CD4^+^/CD8^+^). This model had high value in predicting the occurrence of DVT after thoracoscopic LC resection, with an AUC of 0.934 and an estimated probability of 0.715 (meaning that patients would be categorized as high risk of DVT if the chance were lower than 0.715). The final model for predicting the incidence of DVT 3 days following thoracotomy LC resection was as follows: Logit(*P*) = −2.463 – 0.026(*R* value) – 0.143(*K* value) + 0.402(α angle) + 0.198(D-D) + 0.237(MDA) + 0.409(SOD). This model is also helpful in identifying the occurrence of DVT following thoracotomy LC resection, with an AUC of 0.918 and an estimated probability of 0.686 (meaning that patients would be categorized as high risk of DVT if the chance was lower than 0.686).

### 3.7 Validation of the logistic regression models

A validation cohort analysis was conducted to evaluate the robustness of our logistic regression models. Table 6 contains information regarding the subjects. 86 individuals participated in the validation phase of the investigation.

**Table 6:** Comparison of changes in thromboelastography, coagulation, fibrinolysis, stress response, and immune function indicators at different time points before and after operation in the verification cohort (*n* = 86) (results are represented as the mean value ± SD)

Indicators	Thoracoscopic LC resection (*n* = 43)				Thoracotomy LC resection (*n* = 43)			
	Preoperative	1 day after operation	3 days after operation	5 days after operation	Preoperative	1 day after operation	3 days after operation	5 days after operation
**Thromboelastography indicators**								
*R* value (min)	5.83 ± 1.22	4.93 ± 0.99^a^	5.49 ± 1.11^a,b^	5.79 ± 1.17^b,c^	5.80 ± 1.19	5.22 ± 1.16^a^	4.98 ± 1.03^a^	5.51 ± 1.19^a,c^
*K* value (min)	1.67 ± 0.49	1.22 ± 0.38^a^	1.45 ± 0.43^a,b^	1.56 ± 0.44^b^	1.69 ± 0.52	1.46 ± 0.39^a^	1.21 ± 0.30^a,b^	1.47 ± 0.45^a,c^
α angle (degree)	63.34 ± 4.98	78.32 ± 6.74^a^	72.16 ± 5.38^a,b^	72.19 ± 5.16^a,b^	63.28 ± 5.01	69.41 ± 5.65^a^	75.25 ± 5.90^a,b^	69.53 ± 5.82^a,c^
MA (mm)	67.08 ± 7.03	74.25 ± 7.45^a^	69.78 ± 6.87^a,b^	70.43 ± 7.03^a,b^	67.14 ± 7.09	72.13 ± 7.26^a^	75.76 ± 7.84^a^	69.12 ± 7.51^c^
**Coagulation indicators**								
PT (s)	12.83 ± 1.16	11.01 ± 0.88^a^	11.78 ± 1.04^a,b^	12.15 ± 1.13^a,b^	12.80 ± 1.19	12.57 ± 1.21^a^	10.77 ± 0.98^a,b^	12.48 ± 1.09^a,c^
APTT (s)	30.17 ± 4.09	29.30 ± 3.94^a^	29.87 ± 4.14^b^	30.16 ± 3.99^b^	30.16 ± 4.02	29.91 ± 4.00	29.24 ± 3.57^a,b^	29.96 ± 4.10^c^
INR	1.00 ± 0.09	1.02 ± 0.07	1.02 ± 0.11	1.01 ± 0.12	1.01 ± 0.10	1.01 ± 0.09	1.02 ± 0.07	1.00 ± 0.11
FIB (g/L)	2.79 ± 0.41	4.36 ± 0.49^a^	3.81 ± 0.30^a^	3.77 ± 0.46^a,b^	2.77 ± 0.42	3.41 ± 0.40^a^	4.47 ± 0.55^a,b^	4.36 ± 0.29^a,b^
D-D (mg/L)	0.13 ± 0.03	0.81 ± 0.14^a^	0.46 ± 0.13^a,b^	0.43 ± 0.10^a,b^	0.12 ± 0.02	0.32 ± 0.08^a^	0.77 ± 0.18^a,b^	0.51 ± 0.14^a,b,c^
**Stress response indicators**								
MDA (mmol/mL)	4.34 ± 1.02	7.13 ± 1.14^a^	6.47 ± 1.03^a,b^	5.17 ± 0.97^a,b,c^	4.26 ± 0.99	5.89 ± 0.98^a^	8.65 ± 1.34^a,b^	6.58 ± 1.16^a,b,c^
SOD (U/mL)	84.17 ± 10.64	66.37 ± 8.35^a^	77.04 ± 10.03^a,b^	82.56 ± 10.55^b^	85.32 ± 10.27	75.23 ± 9.64^a^	63.26 ± 6.88^a,b^	73.30 ± 9.18^a,c^
IL-6 (pg/mL)	8.47 ± 1.20	38.15 ± 8.77^a^	23.34 ± 5.29^a,b^	20.44 ± 4.63^a,b^	8.51 ± 1.17	17.95 ± 5.06^a^	36.98 ± 7.43^a,b^	24.04 ± 6.25^a,b,c^
CRP (ng/mL)	10.38 ± 1.98	34.76 ± 7.58^a^	21.43 ± 5.78^a,b^	16.36 ± 5.23^a,b,c^	10.66 ± 2.05	19.53 ± 4.87^a^	33.88 ± 6.86^a,b^	26.40 ± 5.83^a,b,c^
**Immune function indicators**								
CD4^+^ (%)	47.46 ± 4.11	21.47 ± 4.36^a^	28.24 ± 4.73^a,b^	31.69 ± 4.98^a,b^	45.37 ± 3.93	29.20 ± 4.53^a^	20.38 ± 4.17^a,b^	24.36 ± 3.67^a^
CD8^+^ (%)	30.05 ± 2.67	16.64 ± 2.47^a^	18.46 ± 3.05^a,b^	19.93 ± 2.50^a,b^	29.27 ± 2.43	18.96 ± 2.87^a^	16.17 ± 2.44^a^	16.46 ± 2.54^a^
CD4^+^/CD8^+^	1.56 ± 0.14	1.29 ± 0.13^a^	1.53 ± 0.10^b^	1.59 ± 0.12^b^	1.55 ± 0.13	1.54 ± 0.14	1.26 ± 0.11^a,b^	1.48 ± 0.13^c^

**Abbreviation:** *R* value: reaction time value; MA: maximum amplitude; PT: prothrombin time; APTT: activated partial thromboplastin time; FIB: fibrinogen; D-D: D-dimerization; IL-6: interleukin 6; MDA: malondialdehyde; SOD: superoxide dismutase; CRP: C-reactive protein.

**Figure 3:**
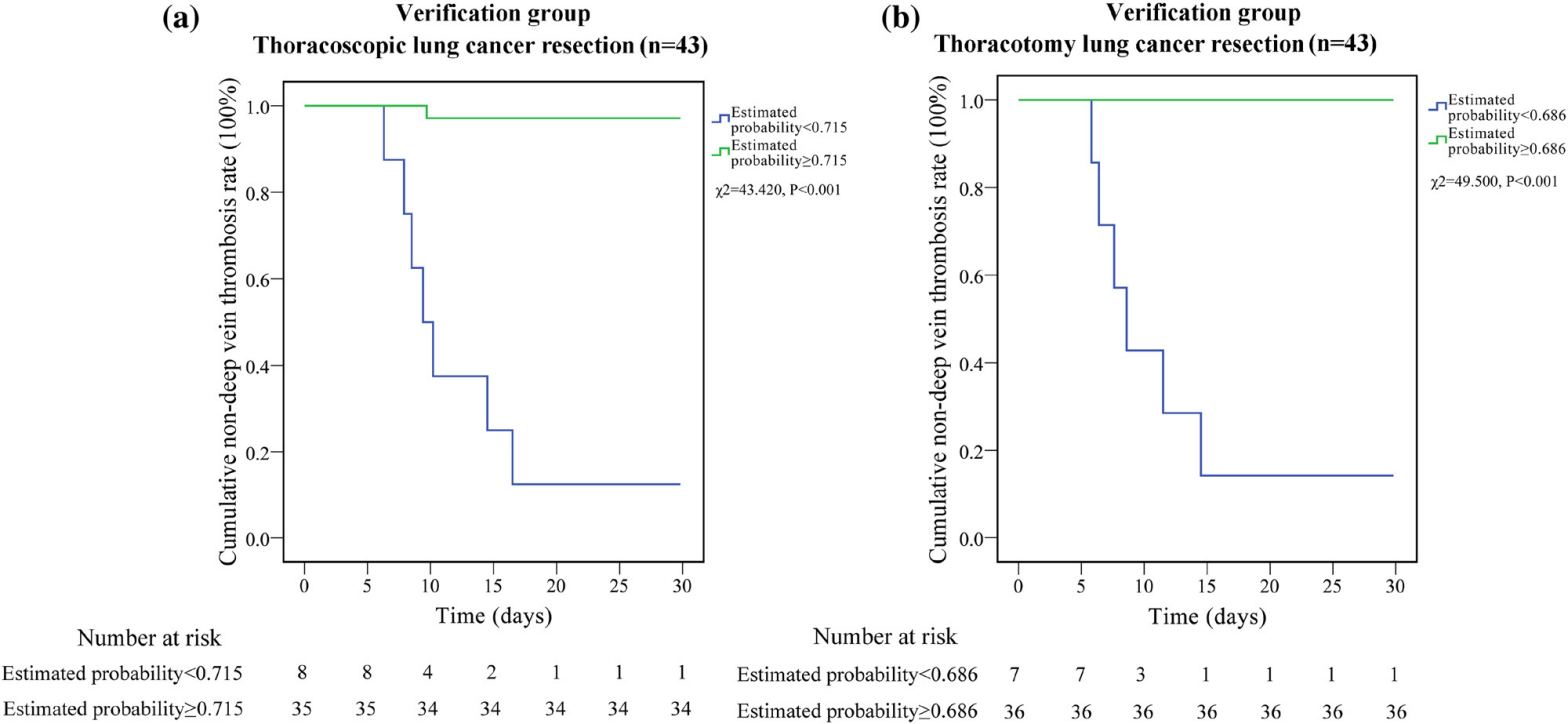
Validation of the logistic regression models in the validation cohort. (a) Comparison of the incidence of DVT between the group of estimated probability ≥0.715 and the group of estimated probability <0.715 in the thoracoscopic LC resection group. (b) Comparison of the incidence of DVT between the group of estimated probability ≥0.686 and the group of estimated probability <0.686 in the thoracotomy LC resection group. DVT: deep venous thrombosis.

**Figure 4:**
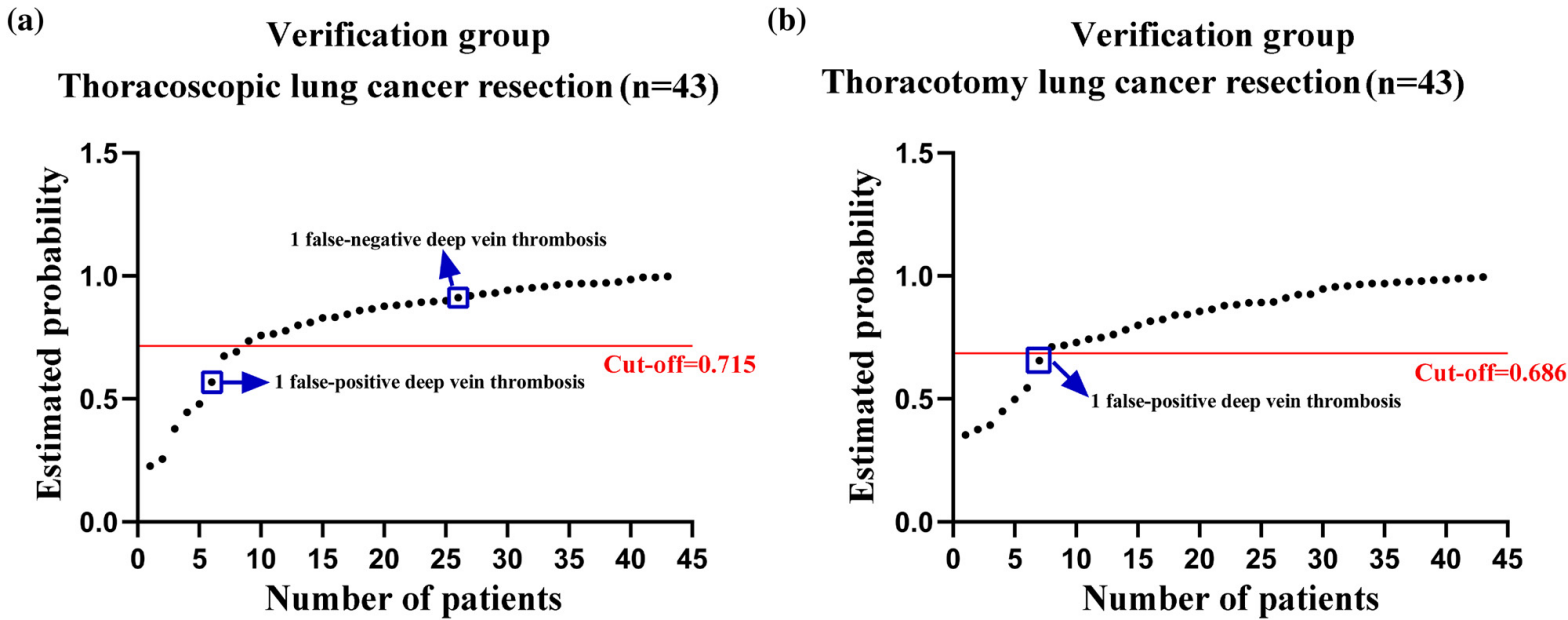
The scatter diagram of the established risk prediction models for the occurrence of DVT after LC resection in the validation cohort. (a) Thoracoscopic LC resection. (b) Thoracotomy LC resection. DVT: deep venous thrombosis.

The calculated probability of thoracoscopic LC resection was 0.715 in one group (*n* = 8) and less than 0.715 in another group (*n* = 35). Outcomes are shown in Figure 3a. Patients with an estimated probability ≥0.715 had a significantly higher incidence of DVT than those with an estimated probability of 0.715 (*χ* ^2^ = 43.420, *P* < 0.001). 7 out of 8 LC patients had probabilities less than 0.715, while 34 out of 35 patients had probabilities greater than 0.715 when using the algorithm. The model’s sensitivity and specificity for predicting DVT after thoracoscopic LC resection were 87.5% and 97.1%, respectively (Figure 4a).

As depicted in Figure 3b, patients undergoing thoracotomy for LC resection were divided into two groups: those with an estimated probability of 0.686 or less (*n* = 7) and those with an estimated probability of 0.687 or greater (*n* = 36). Patients in the group with an estimated probability of 0.686 had a significantly higher incidence of DVT than those in the group with an estimated probability of 0.026 (*χ* ^2^ = 49.500, *P* < 0.001). According to the formula, all the 36 LC patients had odds greater than 0.686, whereas 6 of the 7 LC patients had odds less than 0.686. The model had 85.7% sensitivity and 100% specificity for predicting the presence of DVT after thoracoscopic LC excision (Figure 4b).

## 4 Discussion

LC is still effectively treated with surgery. A comprehensive treatment plan based on surgery has substantially improved the 5 year survival rate and quality of life of patients, particularly those with advanced LC [21]. Current standard surgical procedures for LC resection include thoracotomy and thoracoscopic approaches. The advantages of thoracoscopic surgery, which include less trauma, speedier recovery, and shorter hospital stays, have led to its widespread acceptance [5].

Postoperative complications are frequently one of the most influential factors on the short- and long-term clinical outcomes of patients. Common complications following LC surgery include postoperative infection, pleural effusion, and postoperative DVT, the most dangerous of which is DVT [22]. In previous research, the formation of DVT was attributed to three factors: stagnant venous blood flow, venous endothelial injury, and blood hypercoagulability [23–25]. Among these, venous blood flow is frequently associated with the hemodynamics (*R* value, *K* value, α angle, and MA) of the blood in the vein [25]. Venous endothelial damage is related to stress response and immune function [26,27]. Blood hypercoagulability can be measured by coagulation (PT and APTT) and fibrinolytic (FIB and D-D) functions [27,28]. However, the impact of thoracoscopic and thoracotomy LC resection on patients with postoperative DVT has not been thoroughly investigated. This study compared the factors related to coagulation, fibrinolysis, thromboelastography, stress response, and immune function between the two groups of surgical procedures to determine their impact on postoperative DVT. As a product of fibrin degradation, D-D levels appear to be a useful diagnostic indicator in cases of suspected DVT. Elevated D-D levels can be used as a strong indicator of hypercoagulability because they typically signify an increase in the coagulation and fibrinolysis systems [29]. The findings of this study revealed that the thoracoscopic group had higher D-D levels 1 day after surgery than the thoracotomy group on the third day, suggesting a higher incidence of DVT in the thoracoscopic group. A prior study also suggested that a higher D-D level is associated with a higher chance of developing DVT [30]. Indicators of stress response, such as IL-6, MDA, and SOD, were also examined. The results showed that the thoracoscopic group had higher levels of IL-6 and MDA and lower levels of SOD at 1 day after surgery than the thoracotomy group, which had a similar outcome but on the third day after surgery. This shows that the group who underwent thoracoscopic surgery was at higher risk for DVT. The previous work for IL-6, MDA, and SOD in determining the risk for DVT in patients showed similar findings [31]. Similarly, immune-related markers decreased in the thoracoscopic group 1 day after surgery, while they decreased in the thoracotomy group 3 days after surgery, indicating the LC prognosis. Similar findings have been documented in the literature, where decreased CD4^+^ and CD8^+^ values signify a higher incidence of LC [32].

In thoracoscopic LC resection, the establishment of pneumoperitoneum is crucial to the execution of the procedure and the formation of the surgical field of vision. In recent years, however, it has been reported that the establishment of CO_2_ pneumoperitoneum, operation duration, and patient body position during thoracoscopic surgery may cause significant changes in hemodynamics and coagulation function, thereby promoting thrombus formation [33–35]. Establishment of CO_2_ pneumoperitoneum can significantly increase the diameter of the femoral vein and diminish the venous blood flow velocity of the lower extremities [34]. In addition, pneumoperitoneum can compress the inferior vena cava and iliac veins on both sides, resulting in an increase in venous blood flow resistance and a decrease in blood flow, which will impede venous blood return [35]. Additionally, the pneumoperitoneum has the ability to lift the diaphragm and raise the pressure in the thoracic cavity. As a result, the filling of the heart’s ventricles during diastole is reduced, and the inferior vena cava’s resistance is increased [36]. The aforementioned factors can directly or indirectly obstruct the venous blood of the lower extremities and impede blood return. Consistent with the prior theoretical foundation, the testing cohort in this research found that the incidence of DVT was significantly higher in the thoracoscopic LC resection group than in the thoracotomy LC resection group [35,36]. The risk of DVT was modeled following the two surgical procedures because of the substantial differences in the mechanism of causing DVT between the two.

Based on a comparison of the changes in the laboratory indicators of the patients before the operation and 1 day, 3 days, and 5 days after the operation, the most significant laboratory indicators were incorporated into the logistic regression models for this study. It was discovered that the two final regression models had a prediction accuracy of 87.8 and 80.7% and a specificity of 100% for postoperative DVT in patients with thoracoscopic LC resection and thoracotomy LC resection. In addition, these models were validated in a single validation cohort. The outcomes demonstrated that these two models still had a high level of predictive efficiency (for thoracoscopic LC resection, the false-positive rate was 12.5% and the false-negative rate was 2.9%). For thoracotomy LC resection, the false-positive rate was 14.3% and the false-negative rate was 0%, indicating that these established models can predict DVT in patients after thoracoscopic and thoracotomy LC resection.

In conclusion, the predictive accuracy of postoperative DVT in patients undergoing thoracoscopic and thoracotomy LC resections may be improved with the use of current risk prediction models, which include laboratory indicators for coagulation, fibrinolysis, thromboelastography, stress response, and immune function.

Patients undergoing thoracoscopic or thoracotomy LC resection may benefit from an updated, more accurate risk prediction model for postoperative DVT prediction. Evaluation of DVT-risk and significance of prophylactic measures is still considered to be lacking and future efforts with prospective study models are required. Future investigations should assess postoperative complications using validated scales. However, this investigation was conducted as a prospective case-control study at a single center, which limits its applicability and dissemination. In the future, it is hoped that this model will be further validated in a significant, multi-center, retrospective validation study that will help Chinese patients.

## Supplementary Material

Supplementary Table
