# Supplementary material for: Novel risk prediction models for deep vein thrombosis after thoracotomy and thoracoscopic lung cancer resections, involving coagulation and immune function
Source: Open Life Sci. 2023 May 23;18(1):20220617. doi: 10.1515/biol-2022-0617 (PMC10224613; doi:10.1515/biol-2022-0617)
Supplement: Supplementary Table [file biol-2022-0617-sm.pdf]

Supplementary material

Table S1: Comparison of general information between patients with deep vein thrombosis or not in the testing cohort (n=374)

Indicators	Thoracoscopic lung cancer resection (n=187)				Thoracotomy lung cancer resection (n=187)			
	Non DVT group (*n*=152)	DVT group (*n*=35)	$t/Z/\chi^2$	*P*	Non DVT group (*n*=166)	DVT group (*n*=21)	$t/Z/\chi^2$	*P*
Gender (male/female)	102/50	24/11	0.028	0.868	122/44	15/6	0.041	0.840
Age	54 (26–77)	53 (25–78)	0.126	0.564	55 (27–76)	53 (30–78)	0.098	0.863
BMI (kg/m^2)	24.01±3.21	24.42±3.42	−0.673	0.502	23.79±3.11	22.80±2.99	1.380	0.169
Hypertension (n, %)	41	8	0.249	0.618	38	5	0.004	0.948
Diabetes (n, %)	24	4	0.425	0.514	18	4	1.209	0.272
Hyperlipidemia (n, %)	26	5	0.164	0.686	21	5	1.939	0.164
Anticoagulation immediately after operation (n, %)	77	15	0.432	0.511	76	11	0.326	0.568
Tumor size (cm)	4.1 (1.1–7.4)	4.3 (1.3–7.5)	0.293	0.447	4.4 (1.1–7.7)	4.3 (1.0–7.4)	0.187	0.741
TNM staging (I/II/III)	52/29/71	11/5/19	0.764	0.682	50/37/79	7/5/9	2.666	0.264
Operation time (min)	235.71±35.93	251.20±35.42	−2.305	0.022	194.79±24.72	201.31±26.42	−1.130	0.260
Intraoperative blood loss (ml)	186.92±26.33	197.13±26.85	−2.061	0.041	295.12±78.91	304.90±81.26	−0.533	0.594
Postoperative hospital stay (d)	9.62±1.54	10.15±1.96	−1.739	0.084	14.50±2.16	15.41±2.45	−1.791	0.075
**R value (min)**								
1 days after operation	5.07±1.11	4.11±0.94	4.738	<0.001	5.29±1.10	5.47±1.13	−0.704	0.482
3 days after operation	5.29±1.05	5.56±1.09	−1.362	0.175	5.21±1.15	3.96±0.97	4.768	<0.001
**K value (min)**								
1 days after operation	1.30±0.42	0.71±0.32	7.799	<0.001	1.38±0.40	1.46±0.45	−0.851	0.396
3 days after operation	1.49±0.46	1.38±0.40	1.305	0.193	1.28±0.34	0.75±0.21	6.967	<0.001
**α angle (degree)**								
1 days after operation	75.57±6.23	84.17±6.87	−7.221	<0.001	67.06±5.10	67.51±5.18	−0.380	0.704
3 days after operation	72.99±5.47	74.49±5.49	−1.462	0.146	72.87±5.32	85.96±6.11	−10.445	<0.001
**MA (mm)**								
1 days after operation	74.11±7.03	82.23±7.41	−6.099	<0.001	69.98±7.63	70.60±7.81	−0.350	0.727
3 days after operation	71.01±7.14	70.69±7.08	0.239	0.811	75.48±8.11	87.50±8.36	−6.378	<0.001
**PT (s)**								
1 days after operation	11.44±0.97	8.23±0.87	17.977	<0.001	12.62±1.10	12.80±1.12	−0.705	0.482
3 days after operation	12.01±1.09	12.17±1.12	−0.779	0.437	10.97±0.99	8.39±0.85	11.415	<0.001

(*Continued*)

Table S1: *Continued*

Indicators	Thoracoscopic lung cancer resection (n=187)				Thoracotomy lung cancer resection (n=187)			
	Non DVT group (n=152)	DVT group (n=35)	$t/Z/\chi^2$	P	Non DVT group (n=166)	DVT group (n=21)	$t/Z/\chi^2$	P
**APTT** (s)								
1 days after operation	29.18±4.04	29.66±4.08	−0.633	0.528	29.92±3.98	30.10±4.05	−0.195	0.846
3 days after operation	29.97±4.13	30.08±4.18	−0.142	0.887	29.66±4.46	28.06±4.53	1.546	0.124
**FIB** (g/L)								
1 days after operation	4.11±0.51	6.09±0.62	−19.854	<0.001	3.46±0.43	3.55±0.49	−0.889	0.375
3 days after operation	3.82±0.27	3.88±0.36	−1.109	0.269	4.18±0.49	5.78±0.55	−13.904	<0.001
**D-D** (mg/L)								
1 days after operation	0.57±0.13	1.53±0.16	−37.647	<0.001	0.28±0.08	0.37±0.14	−4.392	<0.001
3 days after operation	0.41±0.14	0.46±0.13	−1.930	0.055	0.73±0.13	1.44±0.19	−22.254	<0.001
**MDA** (mmol/mL)								
1 days after operation	7.19±1.17	8.42±1.32	−5.472	<0.001	5.92±0.87	6.10±1.11	−0.864	0.388
3 days after operation	5.83±1.10	6.04±1.21	−0.999	0.319	8.52±1.09	11.64±1.37	−11.989	<0.001
**SOD** (U/mL)								
1 days after operation	67.22±8.97	67.38±8.36	−0.096	0.923	74.08±9.34	74.26±9.38	−0.083	0.934
3 days after operation	75.09±10.36	75.41±10.87	−0.163	0.871	65.12±7.87	56.93±8.06	4.481	<0.001
**IL-6** (pg/mL)								
1 days after operation	35.22±8.65	44.89±9.23	−5.888	<0.001	19.84±5.28	20.11±5.57	−0.219	0.827
3 days after operation	22.08±5.13	22.13±5.43	−0.051	0.959	34.16±8.32	42.71±8.79	−4.409	<0.001
**CRP** (ng/mL)								
1 days after operation	37.72±7.32	45.68±7.98	−5.702	<0.001	17.47±4.76	17.74±4.93	−0.244	0.808
3 days after operation	18.37±5.03	18.53±5.26	−0.168	0.867	34.67±6.03	35.12±6.38	−0.320	0.749
**$CD4^+$** (%)								
1 days after operation	24.15±4.38	17.41±4.76	8.074	<0.001	28.22±4.29	27.95±4.53	0.270	0.787
3 days after operation	26.98±4.59	27.35±4.87	−0.425	0.671	23.15±4.73	23.33±4.44	−0.165	0.869
**$CD8^+$** (%)								
1 days after operation	17.37±2.40	13.93±2.09	7.821	<0.001	18.13±3.01	17.95±2.94	0.259	0.796
3 days after operation	18.03±3.19	18.08±3.14	−0.084	0.933	17.62±2.49	17.80±2.52	−0.312	0.756
**$CD4^+/CD8^+$**								
1 days after operation	1.39±0.12	1.25±0.11	6.316	<0.001	1.56±0.15	1.55±0.13	0.292	0.771
3 days after operation	1.49±0.14	1.52±0.16	−1.112	0.268	1.31±0.12	1.30±0.10	0.366	0.715

Abbreviation: R value: reaction time value; MA: maximum amplitude value; PT: prothrombin time; APTT: activated partial thromboplastin time; FIB: fibrinogen; D-D: D-dimerization; IL-6: interleukin 6; MDA: malondialdehyde; SOD: superoxide dismutase; CRP: C-reactive protein.
